# Global health worker salary estimates: an econometric analysis of global earnings data

**DOI:** 10.1186/s12962-018-0093-z

**Published:** 2018-03-09

**Authors:** Juliana Serje, Melanie Y. Bertram, Callum Brindley, Jeremy A. Lauer

**Affiliations:** Department of Health Systems Governance and Financing, World Health Organization, Avenue Appia 20, Geneva, Switzerland

## Abstract

**Background:**

Human resources are consistently cited as a leading contributor to health care costs; however the availability of internationally comparable data on health worker earnings for all countries is a challenge for estimating the costs of health care services. This paper describes an econometric model using cross sectional earnings data from the International Labour Organization (ILO) that the World Health Organizations (WHO)-Choosing Interventions that are Cost-effective programme (CHOICE) has used to prepare estimates of health worker earnings (in 2010 USD) for all WHO member states.

**Methods:**

The ILO data contained 324 observations of earnings data across 4 skill levels for 193 countries. Using this data, along with the assumption that data were missing not at random, we used a Heckman two stage selection model to estimate earning data for each of the 4 skill levels for all WHO member states.

**Results:**

It was possible to develop a prediction model for health worker earnings for all countries for which GDP data was available. Health worker earnings vary both within country due to skill level, as well as across countries. As a multiple of GDP per capita, earnings show a negative correlation with GDP—that is lower income countries pay their health workers relatively more than higher income countries.

**Conclusions:**

Limited data on health worker earnings is a limiting factor in estimating the costs of global health programmes. It is hoped that these estimates will support robust health care intervention costings and projections of resources needs over the Sustainable Development Goal period.

**Electronic supplementary material:**

The online version of this article (10.1186/s12962-018-0093-z) contains supplementary material, which is available to authorized users.

## Background

The sustainable development goals (SDGs) represent an ambitious post-2015 agenda. From a health perspective, the broadening of global priorities beyond the millennium development goal (MDG) conditions, as well as the introduction of a target specific to Universal Health Coverage (UHC), indicate growing demands placed on the health system in every country [1, 2]. A background paper for the recent High Level Commission on Health Employment and Economic Growth showed projections indicating an estimated shortfall of approximately 18 million health workers by 2030 [3]. Such projections are an important contributor to the policy dialogue; however, important concerns about the related increase in domestic and external financial resources needed to support these required additional health staff remain unanswered [4]. Human resources are consistently cited as a leading contributor to health care costs, responsible for approximately 57% of total expenditure on health according to unpublished ‘raw’ country data in the Global Health Expenditure Database (GHED) (e.g. uncorrected for on-budget donor assistance) [5, 6] In published studies, human resources have been found to account for between 42% and 46% of total health expenditure [7, 8]. Despite the importance of the contribution of human resources to health financing needs, policy makers and academics are often unable to identify consistent sources of earnings estimates for cost analysis, leading to information gaps in the global policy arena [9].

The demand for the cost analysis of health interventions has increased as governments seek to make informed decisions about how to allocate resources and manage their budgets in low resource settings [10]. Cross country estimates on the cost of health interventions help in this respect both donor agencies such as Gavi and the Global Fund, and international organizations, such as the World Health Organization. Recognizing a need for publicly available information on the costs and cost-effectiveness for health interventions, the WHO-CHOICE programme has made available since 2000, a database of price estimates of various intervention inputs for use in the economic and financial analyses of health care interventions [11].

The availability of internationally comparable data on health worker earnings for all countries has nevertheless persisted as a challenge. This paper describes an econometric model that uses cross sectional occupational wage data from the International Labour Organization [12] that WHO-CHOICE has used to prepare estimates of health worker wages (in 2010 USD) for all WHO member states by skill level. It is hoped that these estimates will support robust health care intervention costings and projections of resources needs over the SDG period.

The aim of this paper is firstly to provide a comparative global database of health worker salary data that can be used in global or regional resource needs assessments. These types of analyses are most useful for multilateral agencies, such as WHO, who provide normative estimates of “global price tags” or for global health initiatives such as GAVI and The Global Fund who make resource allocation decisions at the global level and require a database of prices estimated with a consistent methodology. A secondary use of the data could be at the country level for resource needs assessments where primary data sources are not available; however, we would anticipate this use case to be relatively rare. Finally, a third use case could be global health analysts/researchers undertaking comparative analyses across settings, or in settings for which they do not have access to primary data. We hope that this paper will provoke global health researchers who collect data on prices at the country level to report more disaggregated figures on salaries to allow better validation of these global data sets at the country level in future iterations of this work.

## Methods

### Source of data

Earnings data were retrieved from the ILO. The ILO’s annual wages and retail prices survey collates data on 159 occupations. ILO’s ISCO-08 classifies occupations into 10 major categories and 4 skill levels. The ILO’s ILOSTAT database contained wages data for only 32 countries from 2009 onwards at the time of data extraction. ILO’s older ILOSTA database contains wages data for most of countries between 1998 and 2008.

Wages data were retrieved from the (ILO) wage estimate database [12] for a variety of job titles across countries, and then classified into four skill levels according to ISCO-08 Major Groups [13] (Table 1, The dataset used in the analysis contains, for each country, (a) a pooled data point of monthly wages (in 2010 USD) for the 4 skill levels, (b) GDP per capita for 2010, (c) country income level and (d) WHO region. The pooled data point refers to a sub-sample of the broader set of countries’ occupational wages available in the ILOSTAT database from 1999 to 2011. As many occupations are represented for each of the skill levels in the ILO database, we selected earnings for medical professions wherever possible; For skill Level 4, data extraction focussed on earnings for general physicians, dentists and professional nurses, for Level 3 the data extraction focussed on earnings for medical X-ray technicians, physiotherapists and auxiliary nurses, Level 2 focused on clerks and secretaries and Level 1 on physical labourers (Additional file 1: Table S1 further information in Appendix A). For those countries that do not have wage data available in ILOSTAT, the former ILO database ILOSTA with data up until 2008 is used. A proxy wage value for each skill level was entered using the salary of a representative occupation from the ILO’s annual wages and retail prices survey (“October Inquiry”). A single representative occupation was used because of the complexity and time that would have been involved in calculating an average salary for different occupations at the same skill level corresponding to different years. As often as possible, the same representative occupation was used for all countries. The preferred representative occupations for each skill level were those that were well-defined and found in most countries. These preferred representative occupations as well as a full list of the occupations covered by the ILO October Inquiry are contained in Additional file 1: Appendix A. Data from the most recent year available in ILOSTAT were used in our econometric analysis. In total, 324 observations from 86 countries were available for analysis.

**Table 1:** Definition of ISCO-08 Major skill level groups [13]

ISCO-08 major group	Skill level
Professionals	4
Technicians and associated professionals	3
Clerical support workers	2
Elementary occupations	1

Nearly 75% of the data is from 2006 or more recent. However, there are a number of countries, mainly low-income countries in Africa, whose most recent data is over 10 years old. Additional file 1: Table S2 in Appendix A presents the distribution of available data by year. The wages were observed by skill level using a box pot (Additional file 2: Figure S1, Appendix B), which showed all distributions to be right skewed with a few outliers. As the skill level increases, the right tail becomes longer and more populated, and the number of outliers decreased. The distribution suggests larger wage differentials at the lower skill levels across countries. This is consistent with the literature [14] that has shown higher rates of migration for lower skilled workers due to high disparity in wages across countries. We expect wages to increase by skill level, to different degrees, depending on the region. To confirm this, we use a bar graph with mean wage by region and skill level (Additional file 2: Figure S2, Appendix B). We expect mean wages to have increased over time globally. A bar graph of mean wages over time showed mean wages peaking in 2007, drastically falling in 2008, and slightly recovering thereafter (Additional file 2: Figure S3, Appendix B). The drop between 2007 and 2008 coincides with the global financial crisis, which is the main factor contributing to this decline. The ILO highlights that the link between wages and labour productivity levels had been “broken” prior to the global financial crisis, and market corrections were necessary in order to create a sustainable wage and productivity link [15].

We use the variable Mortality, as a dummy that captures countries with high infant and adult death rates. The 193 Member States have been divided into 5 mortality strata by the WHO, based on their level of child (5q0) and adult male mortality (45q15) as follows: A = Very low child, very low adult; B = Low child, low adult; C = Low child, high adult; D = High child, high adult; E = High child, very high adult. Counties in strata E, D and C are captured by the Mortality dummy. Countries in strata A are captured by a second dummy, called Developed. The 6 WHO regions are also used as dummies in the analysis of this paper (Africa, Eastern Mediterranean, Latin America, Asia, Western Pacific, Europe, and North America), plus a dummy for North America.

### Model specification

Many countries have incomplete data with only a small percentage of the total occupations collected by the ILO, which limits the number of possible representative wages for the four skill levels. We had 324 observations from 86 countries available for analysis. As data are classified into 4 skill levels, 4 observations are sought for each country, corresponding to 772 (193 × 4) data points. Thus, 58% of possible observations were missing. If the data are missing at random, maximum likelihood estimation and multiple imputation techniques are most common, and vast. Multiple imputation methods produces unbiased estimates, even for large percentages of missing values (say < 50%), when at least one auxiliary variable if available to predict the value of Y (i.e. strong correlates of Y are available) [16]. However, multiple imputation methods require that the data must be missing at random, meaning that the probability of observing the variable of interest (Y = wage) can depend other explanatory variables, but not the value of Y [17]. Modelling the missing mechanism becomes imperative, otherwise one must accept a level of bias.

A partial and semi partial correlation matrix was used to investigate the relationship between missing data and possible covariates for estimation (Additional file 2: Table S3, Appendix B). In the partial and semi partial correlations we find that GDP per capita and certain regions are positively correlated with missing data. The non-significance of the semi partial correlation with GDP per capita tells us that in a model to predict the probability the data is missing, the amount by which R^2^ decreases when removing GDP per capita from the model would not be significant. Conversely, we find that variables such as mortality, WHO regions, or year result in a significant change in the R^2^ of such model. Given the substantial amount of missing data (nearly 60%), and the existence of variables that can be used to predict the probability of observing data, the missing data were classified as missing not at random using Rubins classification for missing values [18]. Under this assumption nonresponse mechanisms are considered non-ignorable, and should be modelled.

We consider the missing wages to be caused by sample selection, with the data incidentally truncated, where the probability of observing a missing wage depends on country characteristics (i.e. sample selection depends on a country’s health care system, cultural practices and perhaps economic development).

Due to the truncation of the missing data, omitted variable bias occurs in the sample due to the sample selection of countries in the ILO database. As a result, the endogenous processes that dictate the probability of observing the variable of interest are modelled separately. Heckman’s [19] two-stage sample selection model allows us to remove endogeneity by replacing missing values with data estimated in an auxiliary model, simple regression models can then be used for the wage estimation. This two-step model offers a means of correcting for non-randomly selected samples with copious missing data that characterizes the phenomenon of incidental selection in censored data. Censoring is present here since there are non-observable responses where one can nevertheless observe all of the explanatory variables [20]. As such, the distribution that applies to the observed data is a combination of discrete and continuous distributions as defined by the latent variables which describe the outcome [21]. In the absence of collinearity problems, the full-information maximum likelihood estimator is preferable in terms of robustness, although limited information two-step also gives reasonable results [22]. For this analysis we find that collinearity is an issue because the key variable (mortality) used in the auxiliary model is correlated (0.63) with the key variable (log GDP per capita) used in the wage equation. Thus, we proceed using Heckman’s subsample OLS (Two Part Model): that is, a probit model for selection equation (i.e. the probability of observing the response) and ordinary least squares (OLS) for the wage equation.

Regression equation:

$$y_{1i}*= x_{i} \beta + \varepsilon_{i}$$

Selection equation:

$$y_{2i}*= z_{i} \gamma + U_{i}$$

such that:

$$y_{1i}= y_{1i} * if \;y_{2i}* >0$$

$$y_{1i} = 0\; if\; y_{2i}* \le 0$$

where x is a vector of explanatory variables which determine the wage, and z is a vector of explanatory variables that determine the probability of observing the wage.

In the context of the skill level wages, the explanatory vector x is comprised of the logarithm of GDP per Capita, the skill level (1–4), and a dummy variable for identifying countries with developed health systems (i.e. very low child and adult mortality). Furthermore, z is a vector comprised of dummy variables which identify specific regions where there are high or low levels of response (i.e. Western Pacific, Asia, Eastern Mediterranean), the logarithm of GDP per Capita, and a dummy variable which identifies high levels of both infant and adult mortality in WHO regions. An elevated level of mortality is used to predict the probability of observing the wage because it implies a low level of development, specifically within health systems, which would be associated with poor data collection within the system.

By observing the log wage data in a density plot, we perceived that the wages are not normally distributed, but rather have a distribution resembling the log-normal distribution. In a Jarque–Bera normality test (Additional file 2: Table S4, Appendix B), the log-wages were tested and the hypothesis of normality was not rejected [23]. It is expected that distribution of the error term in an OLS regression of untransformed variables would not necessarily follow a normal distribution. Normality in the distribution of the errors, a crucial assumption for the OLS framework, is thus preserved by implementing the OLS regression equation using the logarithm of earnings.

The wage function was estimated using generalized linear methods (GLM). In this model, the response variable y_i_ is assumed to follow an exponential family distribution, which is assumed to be a nonlinear function of x_i_. The link function specifies the link between the probability distribution of y and explanatory variables x. Thus, specifying a logarithmic link function says how the expected value of the response relates to the linear predictor of explanatory variables. The gamma distribution has a property shared by the log-normal; namely that its variance is proportional to its mean-squared error (i.e. it has a constant coefficient of variation). Within the GLM framework, both the gamma and Gaussian (normal) distributions were tested, however, the parameter for selection bias (calculated as a mills ratio) was found insignificant under the assumption of a Gaussian distribution. Moreover, a limited information maximum likelihood (LIML) estimator was also tested against two alternative models, OLS and GLM (gamma). The parameter estimates for the LIML were similar to that of the GLM because they both use maximum likelihood methods, and thus, the LIML output was omitted. Lastly, because different sub-samples of countries were observed in different years, all the models were compared both with and without a dummy variable for calendar time.

### Reporting of health worker earnings by cadre

Using the classification of health workers suggested by the health workforce projections developed for the Commission on Health Employment and Economic Growth [3], we summarized our resulting estimates by both health worker cadre and regional or country income level to allow comparison to estimates from the Global Health Expenditure Database on human resource expenditure. We calculate mean earnings for each cadre across the income level, where the mean is weighted by country population size. Results are then presented as multiples of mean GDP per capita for the income level.

## Results

### Model implementation

As the first step, the selection process (i.e. the probability of observing the response) was modelled, and the Mills ratio [24] was calculated in Stata version 12. Table 2 indicates the point estimates of the explanatory variables that determine the probability of observing the wage. For example, as can be seen from Table 2, neither the logarithm of GDP per capita or being a developed country significantly affect the probability of observing wages. High mortality, as well as being located within Asia, the Western Pacific or the Eastern Mediterranean region significantly affects the probability of observing skill level wages. Dummy variables for the year when data was observed were introduced at this stage but found to be statistically insignificant.

**Table 2:** Selection models, stage 1

Variable	Probability of a response	Robust standards error	95% confidence interval
Log of GDP per capita	0.0822 (1.61)	0.0516707	− 0.0190972, 0.1834481
Developed	0.0137 (0.08)	0.169712	− 0.3189542, 0.3463045
Western Pacific	− 0.958 *** (− 6.26)	0.1524606	− 1.257184, − 0.659549
Asia	0.498 *** (2.27)	0.2024287	0.1013962, 0.8949019
Eastern Mediterranean	− 0.744 *** (− 4.31)	0.1764809	− 1.089974, − 0.3981816
High mortality	− 0.651*** (− 4.65)	0.1381482	− 0.9214802, − 0.3799491
Constant	− 0.271 (− 0.86)	0.319379	− 0.897074, 0.3548685
Observations	760		

t statistics in parentheses

* p < 0:05, ** p < 0:01, *** p < 0:001

As we only had one observation in time for each country, this prevented the analysis from taking on time series—panel data—considerations (i.e. unobserved fixed effects). We assumed that observations are independent across countries, but dependent within regions, thus applied an adjustment to the variance covariance matrix at this stage. This allows us to use cluster-robust estimates to address any within-group correlation remaining.

The second step corrects for self-selection by incorporating a transformation of these predicted individual probabilities as an additional explanatory variable. Wages are thus estimated using the regression equation based on the Mills ratio, which can be interpreted as the omitted variable bias if the regression were to use the sub-sample consisting only of y_i1_* > 0.

Table 3 shows the beta coefficients for the wage equation corrected for sample selection. The table includes a comparison of 4 different specifications of the wage equation (OLS with and without a year dummy (t), and a GLM, with and without a year dummy variable). The significance of this Mills ratio coefficient provides evidence in favour of the hypothesis that the wages collection model has selection bias, and that the use of a two-part model was indeed necessary. Note that the regression assigns significant increases to the wages based on skill level (1 being the base comparison). The logarithm of GDP per capita and a dummy variable to identify developed nations were both highly significant. Dummy variables for regions and interaction terms were introduced into the regression, although they did not significantly affect the wages. The sign of the coefficient of the dummy variables for calendar year are inconsistent, and including a dummy variable for time renders the selection bias parameter insignificant for the OLS estimation. Otherwise, the variables all have the same signs across models, indicating consistency in the direction of the estimated effects for the regressors.

**Table 3:** Selection Model: 2nd stage

	(OLS) Log (wages)	(OLS, t) Log (wages)	(GLM) Monthly wages (USD 2010)	(GLM, t) Monthly wages (USD 2010)
Log GDP per capita	0.630*** CI (0.5218216, 0.6826666)	0.701*** CI (0.4905892, 0.7463968)	0.474*** CI (0.366835, 0.6134864)	0.773*** CI (0.5477371, 0.9689427)
Skill level 1	0 (.)	0 (.)	0 (.)	0 (.)
Skill level 2	0.514* CI (0.2477793, 0.7426511)	0.537* CI (0.2483299, 0.7399533)	0.462* CI (0.98367, 0.823418)	0.462* CI (0.0457699, 0.6770993)
Skill level 3	0.769** CI (0.5098665, 0.991286)	0.786** CI (0.5129751, 0.9960829)	0.654*** CI (0.3342827, 0.9630529)	0.659*** CI (0.3371977, 0.8379690)
Skill level 4	1.239*** CI (0.959575, 1.480105)	1.261*** CI (0.9675765, 1.48336)	1.364*** CI (0.8282947, 1.900386)	1.364*** CI (0.7097371, 1.404407)
Mills ratio	0.877* CI (− 0.0750366, 0.6274496)	0.379 CI (0.2409532, − 0.9886227	0.596* CI (0.537331, 1.181299)	0.640* CI (0.3342776, 0.908729)
Developed	0.565** CI (0.5011368, 0.9780193)	0.560** CI (0.5030425, 0.9732712)	0.842*** CI (0.4943383, 1.075699)	0.841*** CI (0.3900876, 0.9895371)
Year		− 0.143* CI (0.0988324, 0.5013373)		0.0126 CI (0.0077645, 0.0344916)
Constant	1.036* CI (0.9810699, 2.206689)	25.00835 * CI (− 59.54842, 109.5651)	2.467*** CI (1.65083, 3.049449)	− 22.85 CI − 57.94873, 44.432837)
Observations	320	320	316	316

Confidence intervals (CI) in parentheses

* p < 0.05, ** p < 0.01, *** p < 0.001

**Fig. 1:**
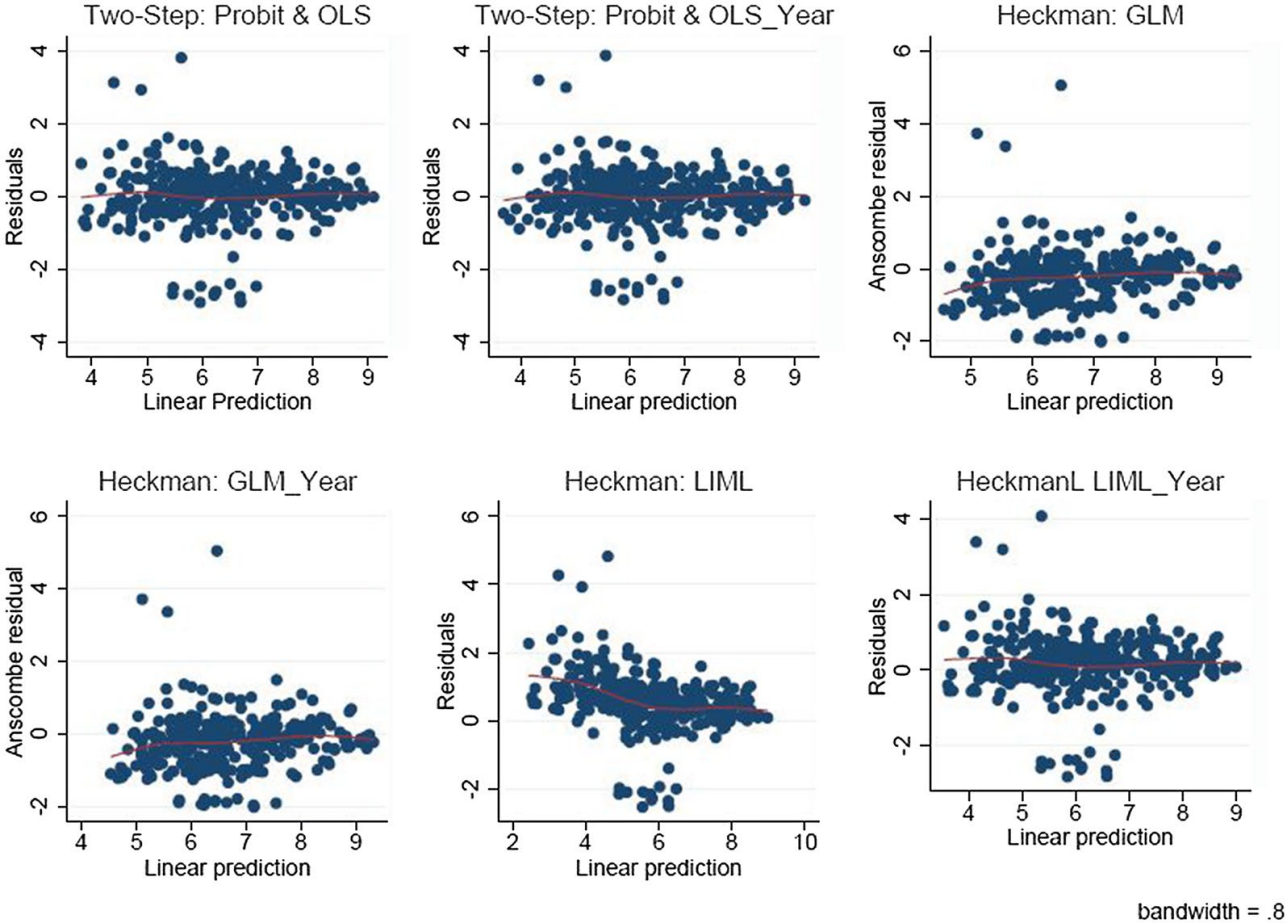
Heckman residual plots

The models were checked for misspecification and goodness of fit using standard regression diagnostic procedures. These include visual inspection of residual plots and statistical assessment for heteroskedasticity. After applying the Huber-White sandwich estimator [25], the residuals appear to be homoskedastic, as shown in Additional file 2: Figure S4, Appendix B. It is hard to convincingly validate these estimates given the amount of missing data. However, model adequacy can be judged from two things. First, the variance of the error term can be used to say something about consistency. As seen by the residual plots (Fig. 1), the Heckman’s OLS estimates without a year dummy produces residuals both closest to zero and with the least amount of variance, and thus, appear to be the most consistent. Second, we plot the distribution of the observed data over the distribution of the predicted values to say something about efficiency (Additional file 2: Figures S5, S6 and S7, Appendix B). The dummy variable for time increased the thickness of the right tail of the distribution for all of the predictions; making the range of the distribution much wider than that of the original data. This indicates that the dummy variable for time is inefficient because it predicts highly implausible wage (> 20,000/month). The same conclusion applies for LIML estimation. The Heckman two-step estimates via OLS produced estimates that were closest to the distribution of the observed data. Estimates were produced for 189 out of the targeted 194 countries. Countries with inadequate information on GDP were unable to be included in the estimation set. A full list of estimated monthly wages data, by country and ISCO-08 skill level is provided in an Annexe to this paper.

### Health worker earnings by income level

The resulting monthly earnings estimates for the three cadres of health workers were converted to annual earnings figures and then expressed in terms of GDP per capita indexes (that is, as multiples of GDP per capita). Expression of annual earnings in terms of units of GDP per capita has the desirable feature that, for projection purposes, per capita wage indexes can be assumed to remain constant, while estimates of GDP per capita change according both to projections of economic growth and to United Nations Population Division projections of population growth.

ILO data on average wages are presented in terms of four professional categories. In this analysis, we assume that the predicted mean of category 4 (equivalent to the second stage of tertiary education) provides a valid estimate of the wages of doctors as specified by the ISCO-08 skill levels. For the wage estimates for nurses and midwives, we use an average of category 4 and category 3 levels (the latter is equivalent to the first stage of tertiary education). For “other health workers”, we use an average of categories 3, 2 and 1. This follows the classification of staff cadres developed in health workforce projections [3] Our resulting estimates are then expressed in terms of GDP per capita indexes (i.e. as multiples of GDP per capita) (Table 4).

**Table 4:** Health worker earnings as a multiple of GDP per capita

World bank income categories	Health worker cadre	Average earnings index (multiple of GDP per capita)
High-income countries	Physicians	1.9
	Nurses and midwives	1.5
	Other health workers	0.9
Upper-middle-income countries	Physicians	2.7
	Nurses and midwives	2.2
	Other health workers	1.3
Lower-middle-income countries	Physicians	5.1
	Nurses and midwives	4.2
	Other health workers	2.4
Lower-income countries	Physicians	7.8
	Nurses and midwives	6.4
	Other health workers	3.7
Global	Physicians	4.4
	Nurses and midwives	3.6
	Other health workers	2.1

**Fig. 2:**
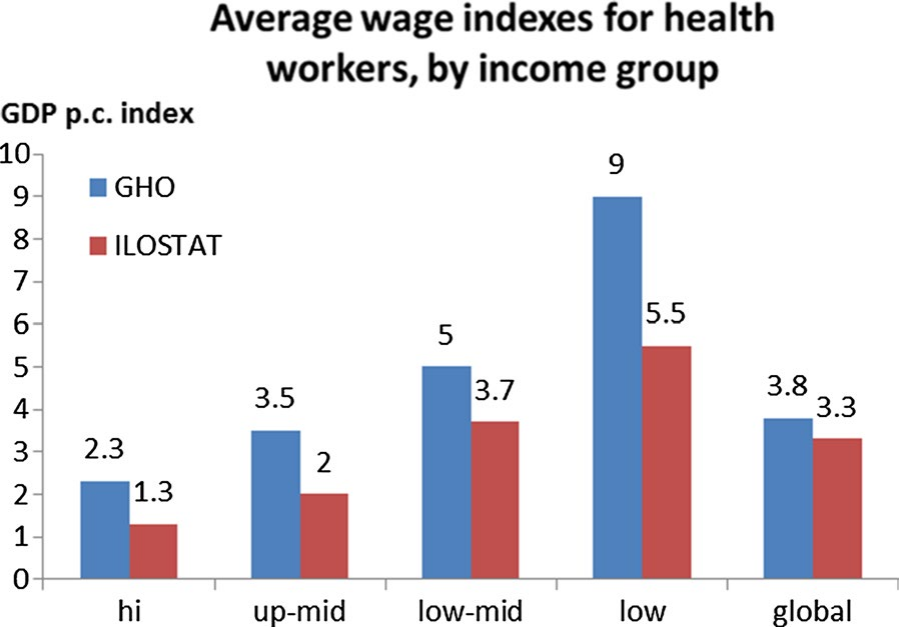
Comparison of average health worker earnings indexes estimated from ILOSTAT and from the Global Health Expenditure Database

For validation, these average health worker wages indexes are compared to alternative estimates of average health worker wages indexes derived from unpublished estimates of earnings from the Global Health Expenditure Database (http://www.who.int/ghed) [6]. These data are drawn from National Health Accounts information used to track health expenditure in the previous year, using a top down costing approach. The side-by-side comparison is shown in Fig. 2. GHED data shows higher expenditure on health workers than our salary estimates from ILO indicate. Both the data published by the ILO and those obtained from the Global Health Expenditure Database are subject to measurement error, and they also both have numerous missing observations [26]. The GHED data includes expenditures that are not included in a more direct measurement of salary as drawn from ILOSTAT. An example of this may be health worker uniforms that are supplied to workers thus are a health expenditure directly related to workforce, but may not be included as remuneration by a worker reporting a salary [27]. However, these two sources of estimates of health worker earnings agree in important respects, showing the same increasing pattern across income levels with the GHO consistently higer than ILO stat. This can be explained firstly by the broader definition of earnings, and secondly by the top down costing approach. In our view, they therefore establish a “plausibility range” of GDP per capita wage indexes for health workers.

## Discussion

When unpublished raw data, that is, uncorrected for on-budget overseas development assistance or exposed to rigorous validity checks, are expressed as a proportion of total health expenditure, human resource costs account for 57% of total health expenditure data. With such a high (nominal) contribution to total health expenditure, the limited data on expected wages by country is a major limiting factor in estimating the financial needs required to achieve the sustainable development goals, and particularly to achieve the target on universal health coverage [28]. This analysis uses existing datasets to estimate health worker wages by country and to examine trends by income level, as well as to provide information for health care planners, analysts and global health donors to use in developing financing projections.

Overall the earnings data show an inverse relationship with income, in that higher-income countries and regions show lower estimated health-worker wage indexes. In other words, the wages of health workers are higher (as a multiple of GDP per capita) in lower-income than in higher-income countries, despite wages in higher-income countries being higher in absolute terms. Both the wages data published by ILO and the aggregate-level data on wages obtained from the Global Health Expenditure Database display many missing observations, and there are in addition multiple potential sources of measurement error. Yet these two sources of estimates agree in important respects and therefore appear to show a plausible range for GDP per capita wages indexes for health workers.

Health workforce wages were first estimated by WHO-CHOICE based on ILO data in 2000 [11]. A comparison of inflated data from the earlier analysis to the more recent ILO data shows some interesting trends in wages over time. Importantly, health worker wages do not appear to have increased in line with inflation in all settings. In low-income countries, health worker wages are 45% lower than those expected due to inflation alone based on 2000 estimates, in upper-middle income countries 10% less and high income countries 30% less, whereas in lower middle-income countries this is not the case, with wages tracking almost exactly with inflation. Regional variations are also present, as in the Western Pacific Region salaries have increased by twice the amount predicted by inflation, in line with proposals that increased salaries might assist worker retention [29], whereas in the Africa Region salaries increased 35% less than the amount expected due to inflation, which may be a contributing factor in the large proportion of health workers in the region who supplement their salaries [30]. This indicates that economic factors affecting relative wages are at play and suggests that regular, high-quality data collection on wages would be useful to better understand the global trends in health worker wages.

The level of missing data in the ILO data set, along with issues around sample selection and truncation of missing data, create a series of limitations with the data analysis. Our best efforts have been made to address the biases found, as described throughout the methodology. With almost 60% of the data missing, the outputs are necessarily heavily modelled, though with strong econometric techniques. Although the dataset is relatively small there is a lot of information captured through the auxiliary variables used in the model which include, mortality rates, GDP per capita, WHO regions, skill level and year of observation, providing additional confidence in the final data set produced. For the purposes of global, normative analyses of resource needs or price tags, we feel confident that the data provide valid and reliable data. At the country level, we would advise local analysts to seek alternative prices to use in their costing analysis until such time as a more complete dataset, both in terms of missingness and coverage of cadres of workers, of the type developed by the ILO statistics division becomes available.

We hope that this paper will provoke global health researchers who collect data on prices at the country level to report more disaggregated figures on salaries to allow better validation of these global data sets at the country level in future iterations of this work.

## Additional files

**Additional file 1: Appendix A.** Summary Statistics and Model Fit Test.

**Additional file 2: Appendix B.** Details of the ILO Data Used.
