# Supplementary material for: Global health worker salary estimates: an econometric analysis of global earnings data
Source: Cost Eff Resour Alloc. 2018 Mar 9;16:10. doi: 10.1186/s12962-018-0093-z (PMC5845154; doi:10.1186/s12962-018-0093-z)
Supplement: Supplementary file 1 — Additional file 1: Appendix A. Summary Statistics and Model Fit Test. [file 12962_2018_93_MOESM1_ESM.docx]

Appendix A

Table S1:

| **Preference as Representative Occupation** | **ISCO-08 Occupation** | **ISCO Code** | **Skill Level** |
| --- | --- | --- | --- |
|  | 11. Coalmining engineer | 2147 | 4 |
|  | 14. Petroleum and natural gas engineer | 2147 | 4 |
|  | 44. Journalist | 2451 | 4 |
|  | 52. Chemical engineer | 2146 | 4 |
|  | 61. Occupational health nurse | 2230 | 4 |
|  | 76. Power distribution and transmission engineer | 2143 | 4 |
|  | 129. Accountant | 2411 | 4 |
|  | 133. Computer programmer | 2132 | 4 |
|  | 138. Computer programmer | 2132 | 4 |
| 3 | 145. Mathematics teacher (third level) | 2310 | 4 |
| 4 | 146. Teacher in languages and literature (third level) | 2310 | 4 |
|  | 147. Teacher in languages and literature (second level) | 2320 | 4 |
|  | 148. Mathematics teacher (second level) | 2320 | 4 |
|  | 149. Technical education teacher (second level) | 2320 | 4 |
| 1 | 152. General physician | 2221 | 4 |
| 2 | 153. Dentist (general) | 2222 | 4 |
|  | 154. Professional nurse (general) | 2230 | 4 |
|  | 15. Petroleum and natural gas extraction technician | 3117 | 3 |
| 5 | 53. Chemistry technician | 3111 | 3 |
|  | 71. Electronics draughtsman | 3118 | 3 |
|  | 72. Electronics engineering technician | 3114 | 3 |
|  | 94. Book-keeper | 3433 | 3 |
|  | 114. Ship's chief engineer | 3141 | 3 |
| 3 | 118. Air transport pilot | 3143 | 3 |
| 4 | 124. Air traffic controller | 3144 | 3 |
|  | 137. Clerk of works | 3112 | 3 |
|  | 155. Auxiliary nurse | 3231 | 3 |
| 2 | 156. Physiotherapist | 3226 | 3 |
| 1 | 157. Medical X-ray technician | 3133 | 3 |
|  | 45. Stenographer-typist | 4111 | 2 |
| 2 | 46. Office clerk | 412/3/4/9 | 2 |
| 2 | 77. Office clerk | 412/3/4/9 | 2 |
|  | 91. Stenographer-typist | 4111 | 2 |
|  | 92. Stock records clerk | 4131 | 2 |
|  | 95. Cash desk cashier | 4211 | 2 |
|  | 97. Hotel receptionist | 4222 | 2 |
|  | 101. Ticket seller (cash desk cashier) | 4211 | 2 |
|  | 102. Railway services supervisor | 4133 | 2 |
|  | 108. Road transport services supervisor | 4133 | 2 |
|  | 119. Flight operations officer | 4133 | 2 |
|  | 120. Airline ground receptionist | 4221 | 2 |
| 4 | 126. Post office counter clerk | 4212 | 2 |
| 3 | 127. Postman | 4142 | 2 |
|  | 128. Telephone switchboard operator | 4223 | 2 |
|  | 130. Stenographer-typist | 4111 | 2 |
|  | 132. Book-keeping machine operator | 4114 | 2 |
| 1 | 131. Bank teller | 4212 | 2 |
|  | 134. Stenographer-typist | 4111 | 2 |
|  | 135. Card- and tape-punching machine operator | 4113 | 2 |
|  | 140. Stenographer-typist | 4111 | 2 |
|  | 141. Card- and tape-punching machine operator | 4113 | 2 |
| 2 | 142. Office clerk | 412/3/4/9 | 2 |
|  | 13. Underground helper, loader | 9311 | 1 |
| 1 | 28. Labourer | 9322 | 1 |
| 1 | 51. Labourer | 9322 | 1 |
| 1 | 59. Labourer | 9322 | 1 |
| 1 | 70. Labourer | 9322 | 1 |
| 1 | 80. Labourer | 9322 | 1 |
| 1 | 90. Labourer | 9312/9313 | 1 |
| 3 | 100. Room attendant or chambermaid | 9132 | 1 |
|  | 104. Railway vehicle loader | 9333 | 1 |
| 5 | 117. Dockworker | 9333 | 1 |
| 4 | 123. Aircraft loader | 9333 | 1 |
| 2 | 144. Refuse collector | 9161 | 1 |

Table S2: Number of Individual Countries Observed by Year

|  | **Most Recent Data No. of Countries** | **Most Recent Data  % of Countries** |
| --- | --- | --- |
| **2010** | **30** | 34% |
| **2009** | **2** | 2% |
| **2008** | **11** | 13% |
| **2007** | **11** | 13% |
| **2006** | **10** | 11% |
| **2005** | **3** | 3% |
| **>2005** | 67 | 77% |
| **2004** | **3** | 3% |
| **2003** | **1** | 1% |
| **2002** | **4** | 5% |
| **2001** | **2** | 2% |
| **2000** | **5** | 6% |
| **1999** | **4** | 5% |
