# Supplementary material for: Global health worker salary estimates: an econometric analysis of global earnings data
Source: Cost Eff Resour Alloc. 2018 Mar 9;16:10. doi: 10.1186/s12962-018-0093-z (PMC5845154; doi:10.1186/s12962-018-0093-z)
Supplement: Supplementary file 2 — Additional file 2: Appendix B. Details of the ILO Data Used. [file 12962_2018_93_MOESM2_ESM.docx]

**Appendix B**

Figure S1: Median Wage by Skill Level


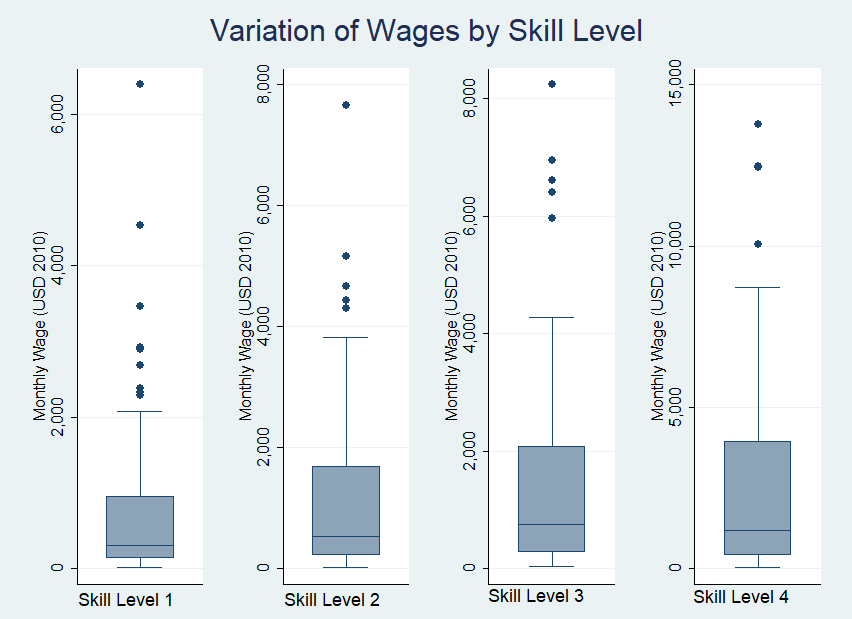


Figure S2: Mean Wage by Region and Skill Level


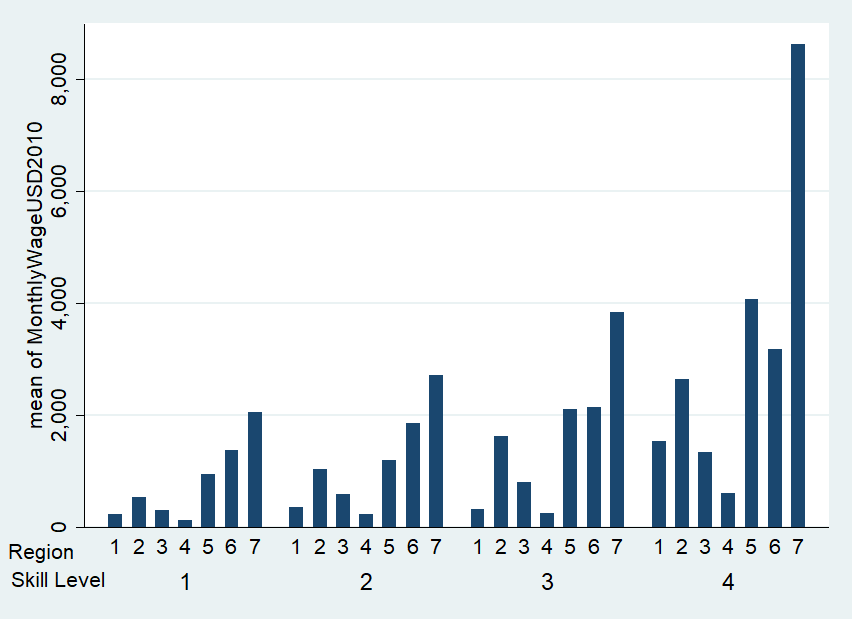


Note that Region 1 is Africa, Region 2 is Eastern Mediterranean, Region 3 is Latin America, Region 4 is Asia, Region 5 is Western Pacific, Region 6 is Europe, and Region 7 is North America.

Figure S3: Mean Wage By Year


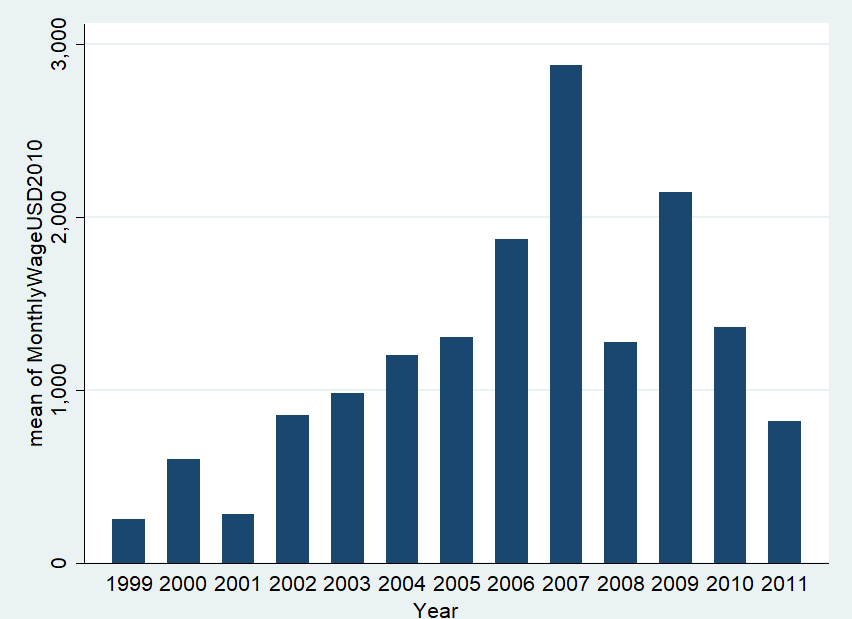


Figure S4: Heckman Residual plots


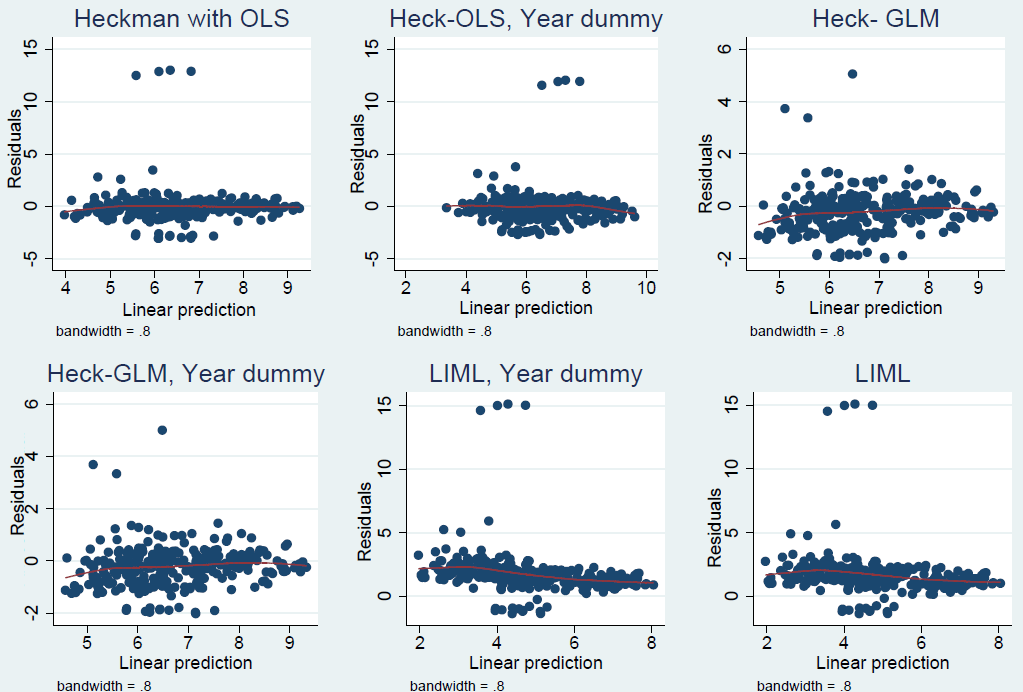


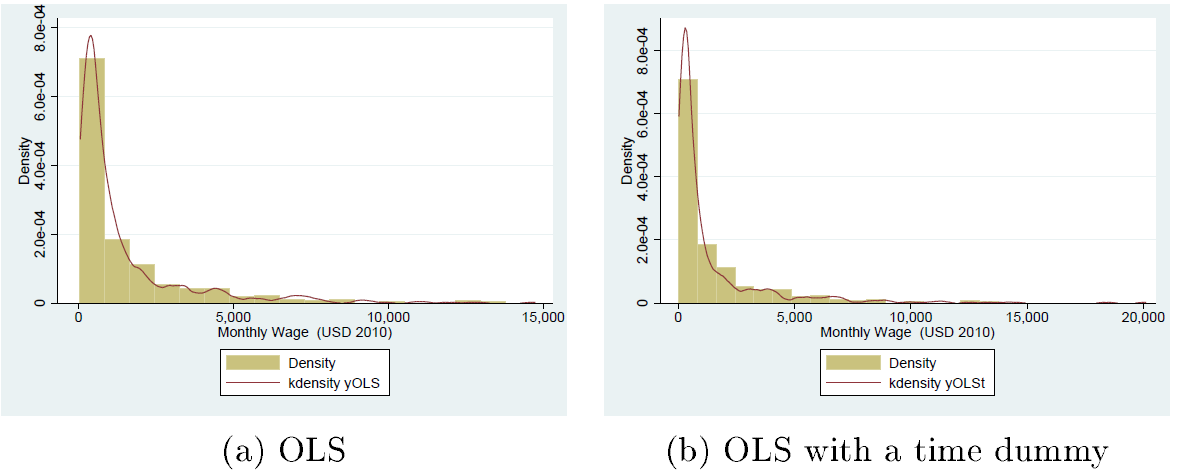
Figure S5: Distribution of the Observed and Predicted Data Using OLS

Note that the true data is represented by the density histogram, while the predicted distribution is indicated by the red line


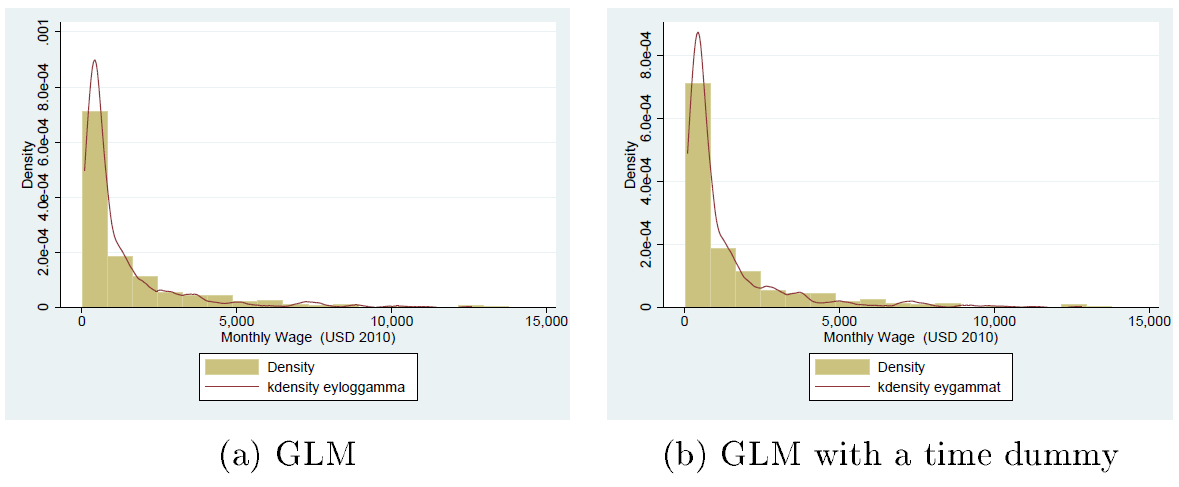
Figure S6: Distribution of The Observed and Predicted Data Using GLM

Note that the true data is represented by the density histogram, while the predicted distribution is indicated by the red line.


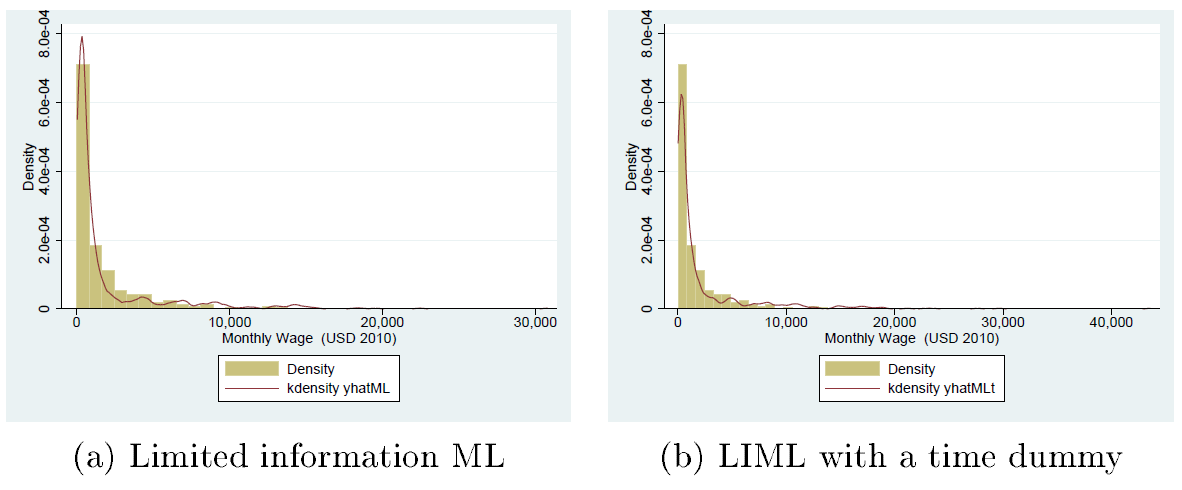
Figure S7: Distribution of The Observed and Predicted Data Using LIML

Note that the true data is represented by the density histogram, while the predicted distribution is indicated by the red line.

Table S3: Partial and Semi-partial Correlation Matrix of Missing Wage

|  | Partial  Corr. | Semipartial  Corr. | Partial  Corr.^2 | Semipartial  Corr.^2 | Significance  Value |
| --- | --- | --- | --- | --- | --- |
| **Variable** |  |  |  |  |  |
| Monthly GDP per cap (2010 USD) | 0.0108 | 0.008 | 0.0001 | 0.0001 | 0.7668 |
| Year | -0.5909 | -0.544 | 0.3491 | 0.296 | 0 |
| Mortality | -0.1239 | -0.0927 | 0.0153 | 0.0086 | 0.0006 |
| Africa | -0.082 | -0.0611 | 0.0067 | 0.0037 | 0.0237 |
| Eastern Mediterranean | -0.098 | -0.0731 | 0.0096 | 0.0053 | 0.0068 |
| Latin America | -0.0537 | -0.0399 | 0.0029 | 0.0016 | 0.1389 |
| Asia | -0.0279 | -0.0208 | 0.0008 | 0.0004 | 0.4417 |
| Western Pacific Region | -0.11 | -0.0822 | 0.0121 | 0.0068 | 0.0024 |
| Europe | -0.0591 | -0.044 | 0.0035 | 0.0019 | 0.1035 |
| North America | 0.0059 | 0.0044 | 0 | 0 | 0.8709 |
| Developed | 0.0372 | 0.0277 | 0.0014 | 0.0008 | 0.3054 |

Table S4: Skewness/Kurtosis tests for Normality

| Variable | Observations | Prb(Skewness) | Prb(Krutosis) | adj chi2(2) | Prob>chi2 |
| --- | --- | --- | --- | --- | --- |
| Log Wage | 320 | 0.2414 | 0.0043 | 8.80 | 0.0123 |

Underlying basis for model selection

Limited dependent variables are commonly missing in social and health data. The structure of the missing data needs to be considered if the underlying distribution of the observed data is to be used to predict missing values. The possibilities are truncation, censoring, or a combination of both, commonly known as sample selection or incidental truncation [11]. In a truncated distribution, only the distribution above or below some value is captured in data collection. For example, if only wages above a or below certain value were captured. Thus, a truncated distribution is simply a part of a larger, not truncated distribution. When data are censored, all observations/events are observed but the values observed within a certain range are all transformed to, or reported as, a single value. The third case, sample selection, occurs when the omission of a data point is not the surveyor’s choice, but rather the data being observed depends on some characteristic of the subject being observed. Humphreys [1] summarizes literature on the avenues available for econometric approaches for estimation of limited dependent variables in these three scenarios (Diagram 1, Appendix A).

Diagram S1: Guide for Model Selection


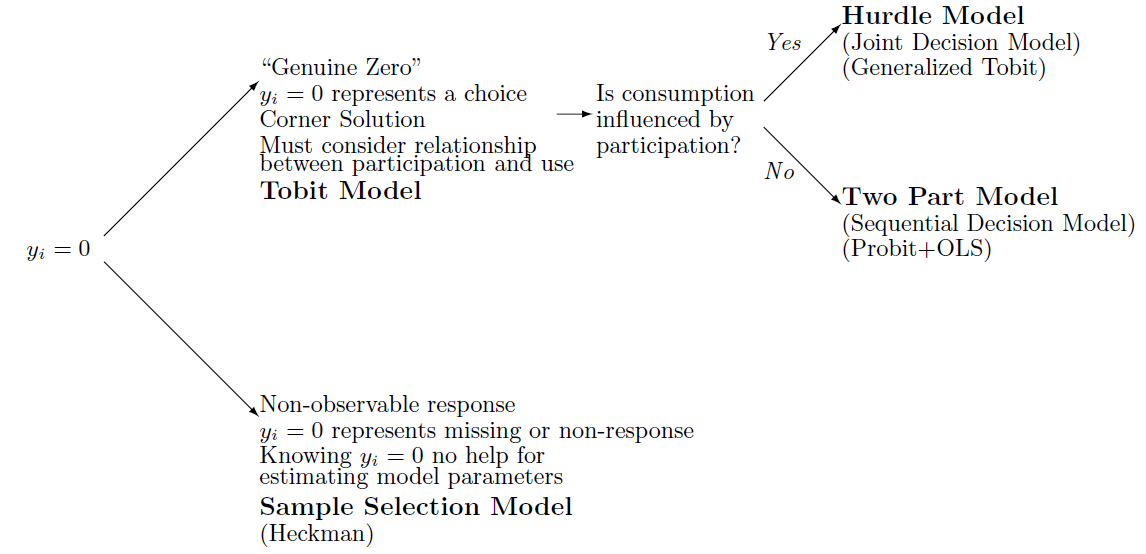


Source:                    

The issues of wage estimation and sample selection have been widely discussed by Heckman in various papers (including 1974, 1976, 1978, 1979). Heckman reviewed estimation of wages for women given the absence of data for those not in the labour force, as well as the effects of union membership on wages[2]. The first case demonstrates sample selection in wage estimation; a model that analyses outcome data (i.e. market wage), using a binary explanatory variable for labour force participation, does not capture the characteristics of women not in the labour force . The second case showcases a treatment effect model; the model for wage estimation making use of outcome data for both union and non-union workers. The models share important similarities; (1) the sample being inferred was not generated randomly; (2) the binary explanatory variable was endogenous; and (3) sample selection or incidental truncation must be considered in the evaluation of the impact of such a dummy variable [3]. The key difference in the two cases of sample selection is that for the former, we only observe the binary explanatory variable, when there is participation ( D=1, and not when D=0 ) . Switching regressions, matching, and instrumental variables models are commonly used to analyse treatment effects in non-experimental research with observational data (i.e. union membership data) given issues of self-selection when there is non-random allocation of subjects to control groups, but both D=1 and D=0 are observed. The literature for assessing models for program/treatment and other types of effects in experimental and nonexperimental contexts has been widely discussed in economics and social science literature [4-7]. On the other hand, when there is Sample Selection Bias, and the variable of interest is truncated (i.e. the wage is not observed for those in the sample where the value of the selection variable == 0), the researcher is faced with a problem of omitted variable bias, and the endogenous processes that dictates the probability of observing the variable of interest should be modelled separately.

1. Humphreys B: **Dealing with Zeros in Economic Data.** (University of Alberta DoE ed. Alberta, Canada; 2013.

2. Heckman JJ: **Sample selection bias as a specification error.** *Econometrica* 1979, **47:**153-161.

3. Guo S, Fraser Mw: *Propensity Score Analysis | SAGE Publications Inc.* Second Edition edn. Thousand Oaks, California: Sage Publications; 2014.

4. Heckman JJ, Richhard R: **Alternative Methods for Solving the Problem of Selection Bias in Evaluating the Impact of Treatments on Outcomes | SpringerLink.** In *Drawing Inferences from Self-Selected Samples.* Edited by Wainer H. New York: SpringerLink; 1989

5. Heckman JJ, Hotz VJ: **Choosing among Alternative Nonexperimental Methods for Estimating the Impact of Social Programs: The Case of Manpower Training.** *Journal of the American Statistical Assoication* 1987, **84:**862-874.

6. Winship C, Mare RD: **Models for Sample Selection Bias.** *Annual Review of Sociology* 1992, **18:**327-350.

7. Ashenfelter O: **Estimating the Effect of Training Programs on Earnings.** *The Review of Economics and Statistics* 1978, **60:**47-57.
